# Research on the Dynamics of Cold Atoms Under Non-Equilibrium Dissipation

**DOI:** 10.3390/e28080890

**Published:** 2026-08-07

**Authors:** Yifan Gao, Yanhang Chen, Shuyu Dai, Bo Cui

**Affiliations:** 1 School of Physics, Dalian University of Technology, Dalian 116024, China; gaoyifan@mail.dlut.edu.cn; 2 Leicester International Institute, Dalian University of Technology, Panjin 124221, China; 3 Key Laboratory of Materials Modification by Laser, Ion and Electron Beams (Ministry of Education), School of Physics, Dalian University of Technology, Dalian 116024, China; 4 School of General Education, Dalian University of Technology, Panjin 124221, China

**Keywords:** non-hermitian hamiltonian, cold atoms, imbalanced dissipation, magnetic flux

## Abstract

We investigate the dissipative dynamics of a one-dimensional sawtooth-shaped Bose–Hubbard model subjected to an external magnetic flux and staggered single-particle dissipation. By combining the Lindblad master equation with a mean-field decoupling and further reducing the dynamics to an effective three-site model, we derive the nonlinear evolution equations that govern the system. Our results reveal that the magnetic flux, acting through the next-nearest-neighbor hopping, determines the preferential direction of particle flow, while the imbalance in dissipation forces the steady-state population to accumulate at lattice sites with weaker loss. Furthermore, we find that two-particle dissipation accelerates the relaxation process when it becomes negative (i.e., gain), whereas positive two-particle loss suppresses localization. These findings demonstrate that directional localization and relaxation dynamics can be controlled by the sign of the next-nearest-neighbor hopping t′ and the magnetic phase, providing a tunable scheme for engineering dissipative quantum states in optical lattices.

## 1. Introduction

The Bose–Hubbard model is a canonical framework for studying strongly correlated quantum systems [1,2], and has played a key role in understanding phenomena such as quantum phase transitions, superfluidity, and localization in ultracold atomic gases and optical lattices [3,4,5]. Traditionally, this model has been developed within the framework of the Hermitian Hamiltonian, where the interaction between field interactions and particle transitions leads to the well-known superfluid-to-Mott insulator transition [6,7]. Over the past two decades, the experimental progress of ulcerated atomic systems has made it possible to precisely control these parameters [8,9], and has expanded the experimental implementation of the three-source subsystem, providing a universal platform for exploring the rich physics of the Bose–Hubbard model.

Existing works largely focus on uniform loss, balanced gain-loss, or topological non-Hermitian effects. However, in cold-atom setups, local dissipation is widely achievable via focused beams, state-selective addressing, or controlled two-/three-body losses. Hence, a sublattice-selective loss bias—acting on alternating sites—merits independent investigation beyond mere extensions of uniform cases.

Such odd–even dissipation breaks discrete translation symmetry by one lattice constant, potentially inducing non-Hermitian phase transitions, reshaping steady-state correlations, or stabilizing exotic non-equilibrium phases. Thus, this bias is not only experimentally relevant but also theoretically essential for connecting non-Hermitian frameworks to realistic quantum-gas platforms.

Nowadays, the latest developments in non-Hermitian quantum mechanics have expanded the scope of Bose–Hubbard models to include dissipative and open systems, where non-Hermitian terms describe gain, loss, or other dissipative processes. Non-hermitian systems exhibit unique phenomena that are not directly similar to Hermitian physics, such as non-Hermitian skin effects (NHSEs) [10,11,12,13,14,15], Exceptional Points (EPs) [16,17,18,19], and non-Hermitian topological phases [20,21,22,23]. These characteristics have aroused great interest in theoretical and experimental research, leading to new insights into the dynamics and steady state of open quantum systems.

However, most existing studies are restricted to uniform or global dissipation, which inadequately captures local and non-equilibrium effects in realistic experiments. In this work, we investigate a non-Hermitian Bose–Hubbard model on a one-dimensional zigzag lattice, focusing on asymmetric dissipation between odd and even sublattice sites. This dissipative bias, in conjunction with an external magnetic flux, gives rise to emergent directional dynamics and enhanced localization. Specifically, we find that the interplay between dissipation imbalance and flux can reverse the evolution preference and induce self-trapping. Meanwhile, sites with stronger loss exhibit lower occupancy; for sufficiently large bias, particles become predominantly confined to the weakly dissipative sublattice. We further examine two-body dissipation, which introduces additional dynamical complexity. Numerical simulations on a reduced model corroborate our theoretical analysis. Our results contribute to non-Hermitian quantum many-body physics and highlight the tunability of quantum states via non-uniform dissipation and external fields. The paper is organized as follows: Section 2 presents the theoretical framework incorporating dissipative bias and synthetic magnetic flux. Section 3 analyzes emergent dynamical phenomena induced by the bias. Section 4 outlines the simplified model and numerical results for various loss imbalances. Section 5 concludes with a discussion of broader implications for quantum science and technology.

## 2. Models and Theoretical Methods

**Figure 1:**
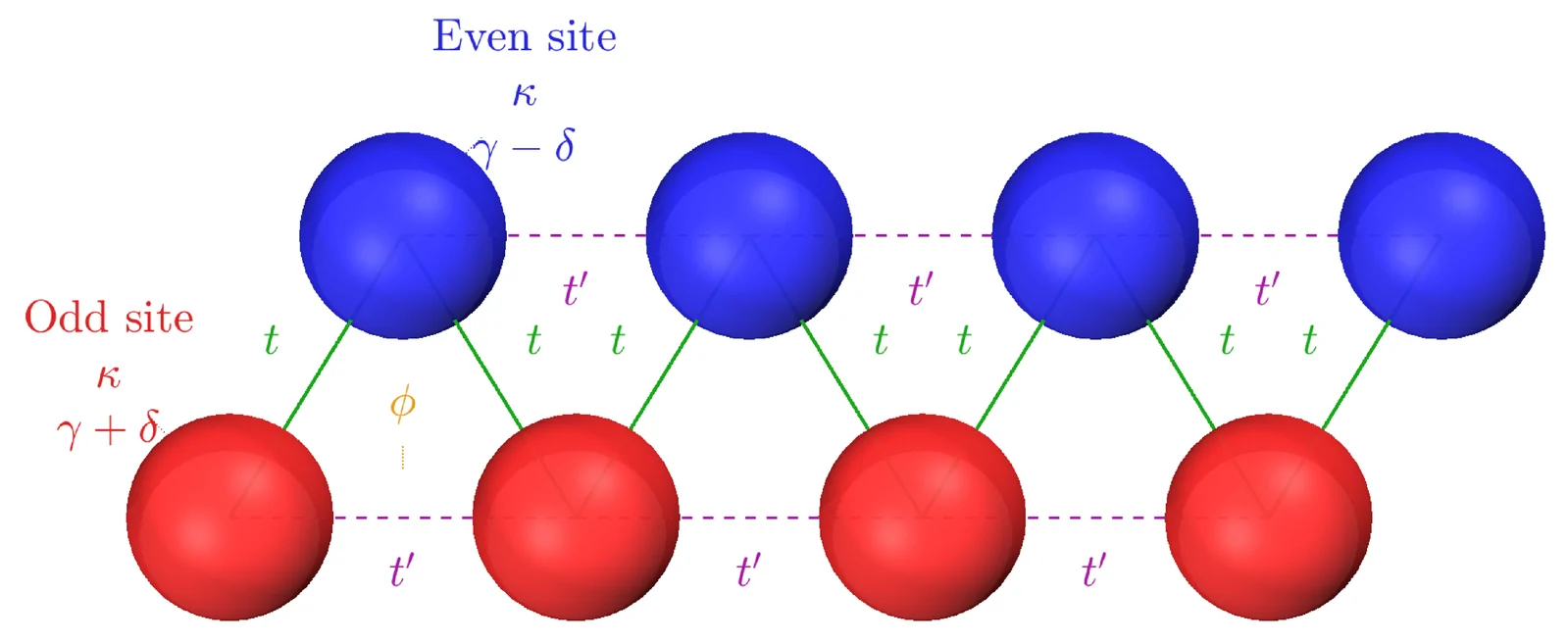
The figure shows a one-dimensional sawtooth-shaped non-Hermitian Bose–Hubbard model under the combined effect of dissipation and transition strength. The transition strength between nearest-neighbor atoms is t, and the transition strength between the next-nearest neighbor is t′. Furthermore, due to the difference in the positions of the atoms at odd and even lattice points, the single-particle dissipation γ±δ will also be manifested. Finally, all the atoms will be subject to the double-particle dissipation of κ. The magnetic flux ϕ passing through the model is also introduced.

In this study, we consider a one-dimensional sawtooth-shaped boson chain composed of atoms in a Bose–Hubbard lattice [24], where interactions and non-uniform dissipation exist, as shown in Figure 1. Naturally, we have also taken the transitions between particles into account, including the transition intensity t between adjacent particles and the transition intensity t′ between the next adjacent particles. And on this basis, we also consider using the external non-zero magnetic flux ϕ to regulate the entire Bose–Hubbard lattice.

On this basis, we can write the Hamiltonian of the boson chain system at this time as

(1) $$\begin{array}{cc} \hat{H} & =-\sum_{j=1}^{L/2-1}\left(t^{\prime }e^{i\phi }{\hat{a}}_{2j+2}^{†}{\hat{a}}_{2j}+t^{\prime }e^{-i\phi }{\hat{a}}_{2j+1}^{†}{\hat{a}}_{2j-1}+H.c.\right)\\ \; & \;-\sum_{j=1}^{L-1}t\left({\hat{a}}_{j+1}^{†}{\hat{a}}_{j}+H.c.\right)-U\sum_{j=1}^{L}{\hat{a}}_{j}^{†}{\hat{a}}_{j}^{†}{\hat{a}}_{j}{\hat{a}}_{j} \end{array}$$

where ${\hat{a}}_{j}$ represents the boson annihilation operator at site j, and t and t′ respectively represent the strengths of the nearest-neighbor and next-nearest-neighbor transitions. U represents the strength of multi-body interactions, and L is the number of optical lattices.

Based on the previously considered non-uniform dissipation between single particles and uniform dissipation between two particles, we can write the dominant dynamic equation of the system according to the Lindblad master equation [6,25,26]

(2) $$\frac{d\hat{\rho }}{dt}=-i\left[\hat{H},\hat{\rho }\right]+\sum_{j}\left(\left[\gamma -{\left(-1\right)}^{j}\delta \right]D\left[{\hat{a}}_{j}\right]\hat{\rho }\right)+\sum_{j}\kappa D\left[{\hat{a}}_{j}{\hat{a}}_{j}\right]\hat{\rho }$$

where $\hat{\rho }$ is the density matrix of the system, and $D\left[L\right]\hat{\rho }$ is the Lindblad superoperator representing the dissipation of bosons:

(3) $$D\left[L\right]\hat{\rho }=L\hat{\rho }L^{†}-\frac{1}{2}\left\{L^{†}L,\hat{\rho }\right\}.$$

Meanwhile, define $\langle a_{j}\rangle \equiv \mathrm{Tr}\left(\rho a_{j}\right)$. Then, the master equation becomes

(4) $$\begin{array}{cc} \frac{d}{dt}\langle {\hat{a}}_{j}\rangle & =it^{\prime }e^{{\left(-1\right)}^{j+1}i\phi }\langle {\hat{a}}_{j+2}\rangle +it^{\prime }e^{{\left(-1\right)}^{j}i\phi }\langle {\hat{a}}_{j-2}\rangle +it\left(\langle {\hat{a}}_{j-1}\rangle +\langle {\hat{a}}_{j+1}\rangle \right)\\ \; & \;+i\left(2U-\frac{\kappa }{2}\right){\left|\langle a_{j}\rangle \right|}^{2}\langle {\hat{a}}_{j}\rangle -\frac{1}{2}\left[\gamma -{\left(-1\right)}^{j}\delta \right]\langle {\hat{a}}_{j}\rangle , \end{array}$$

In addition to the on-site interaction, the equation includes both nearest-neighbor (NN) and next-nearest-neighbor (NNN) hopping processes. The corresponding hopping amplitudes are site-independent and take the uniform values *t* and $t^{\prime }$, respectively. In addition, the equation also contains the dissipation of single particles and that of two particles. Taking these factors into account constitutes the current dynamic equation. In our subsequent simulation of the system, the system we study will include a large number of atoms, so the differences between each atom will become very small. Therefore, in the above process of describing the system dynamics, we used the average field approximation theory [27], which is mainly reflected in $<a^{†}aa>∼<a^{†}a><a>∼{\left|a\right|}^{2}<a>$.

The relative density distribution directly reflects the degree of orderliness of the system. In the superfluid–Mott insulator phase transition, the uniform density corresponds to the Mott state, while the density correlation of oscillation attenuation indicates the superfluid state [8]. Density fluctuations contain interaction information: strong repulsive forces (such as U in Hubbard’s model) suppress density fluctuations, while the density correlation of neighboring lattice points can reveal strange quantum states such as D-wave superconductivity [28].

Therefore, in our current study, we will define the relative density $p_{j}$ of lattice points to characterize the evolution of the system [29,30]. Its definition is

(5) $$p_{j}=\frac{\langle a_{j}^{†}a_{j}\rangle }{\sum_{j}\langle a_{j}^{†}a_{j}\rangle }$$

It can be seen that under such a definition, if we plot the relationship between the relative density and time, we can intuitively understand the relationship between the state of the system and the evolution over time. However, the relative density of the grid points alone cannot represent the number of system states. Based on this, we can continue to define the standard deviation of the relative density on the grid points for observation. Its definition is

(6) $$\sigma^{2}=\sum_{j=1}^{N}{\left(p_{j}-1/N\right)}^{2}$$

After having the standard deviation, we only need to plot the variation relationship of the standard deviation with time to see the state density of the system at that time. Not only that, because of the relationship between the standard deviation and time, we can also see the stability of the system. If the change in the standard deviation over time is small, then the system at this time is relatively stable; otherwise, the system is relatively unstable.

## 3. Results and Discussions

Traditional research on cold atom dissipation mainly focuses on energy dissipation and quantum decoherence caused by irreversible processes such as spontaneous emission, collision and environmental noise [31,32,33]. The core goal is to suppress these effects to improve the coherence and stability of the system. The theoretical basis is mainly quantum master equations and open system dynamics. Non-hermitian dissipation research, on the other hand, transforms traditional dissipation into a controllable quantum control method by meticulously designing a balance between gain and dissipation. Therefore, in our current research, we will focus on the balance control of dissipation and gain.

### 3.1. Analysis of the Influence of Introducing External Magnetic Flux on System Dissipation

Now, we explore the influence of various parameters on the evolution of quantum states and consider the system at odd grid points (even grid points only differ by one phase). When the initial states of the system are located on odd-numbered lattice sites, the evolution (Equation (4)) becomes

(7) $$\begin{array}{cc} \frac{d}{dt}\langle {\hat{a}}_{j}\rangle & =it^{\prime }e^{i\phi }\langle {\hat{a}}_{j+2}\rangle +it^{\prime }e^{-i\phi }\langle {\hat{a}}_{j-2}\rangle +it\langle {\hat{a}}_{j-1}\rangle +it\langle {\hat{a}}_{j+1}\rangle \\ \; & \;+2iU{\left|a_{j}\right|}^{2}\langle {\hat{a}}_{j}\rangle -\frac{1}{2}\left(\gamma +\delta \right)\langle {\hat{a}}_{j}\rangle -\frac{\kappa }{2}{\left|a_{j}\right|}^{2}\langle {\hat{a}}_{j}\rangle . \end{array}$$

We know that the regulatory effect of the magnetic field is periodic. That is to say, when the intensity of the magnetic field increases from 0 to 2π the evolution of the system will also exhibit periodicity. However, we can observe that within the range of 0 to 2π, due to the coupling between the magnetic field and the next-nearest-neighbor transition, it is possible to change the evolution state of the system by adjusting the magnetic field. Taking ϕ = π/2 as an example, suppose the initial state of the system is at an odd grid point (here, we take 31), then the evolution equation of the system is

(8) $$\begin{array}{cc} \frac{d}{dt}\langle {\hat{a}}_{j}\rangle & =-t^{\prime }\langle {\hat{a}}_{j+2}\rangle +t^{\prime }\langle {\hat{a}}_{j-2}\rangle +it\langle {\hat{a}}_{j-1}\rangle +it\langle {\hat{a}}_{j+1}\rangle +2iu{\left|a_{j}\right|}^{2}\langle {\hat{a}}_{j}\rangle . \end{array}$$

**Figure 2:**
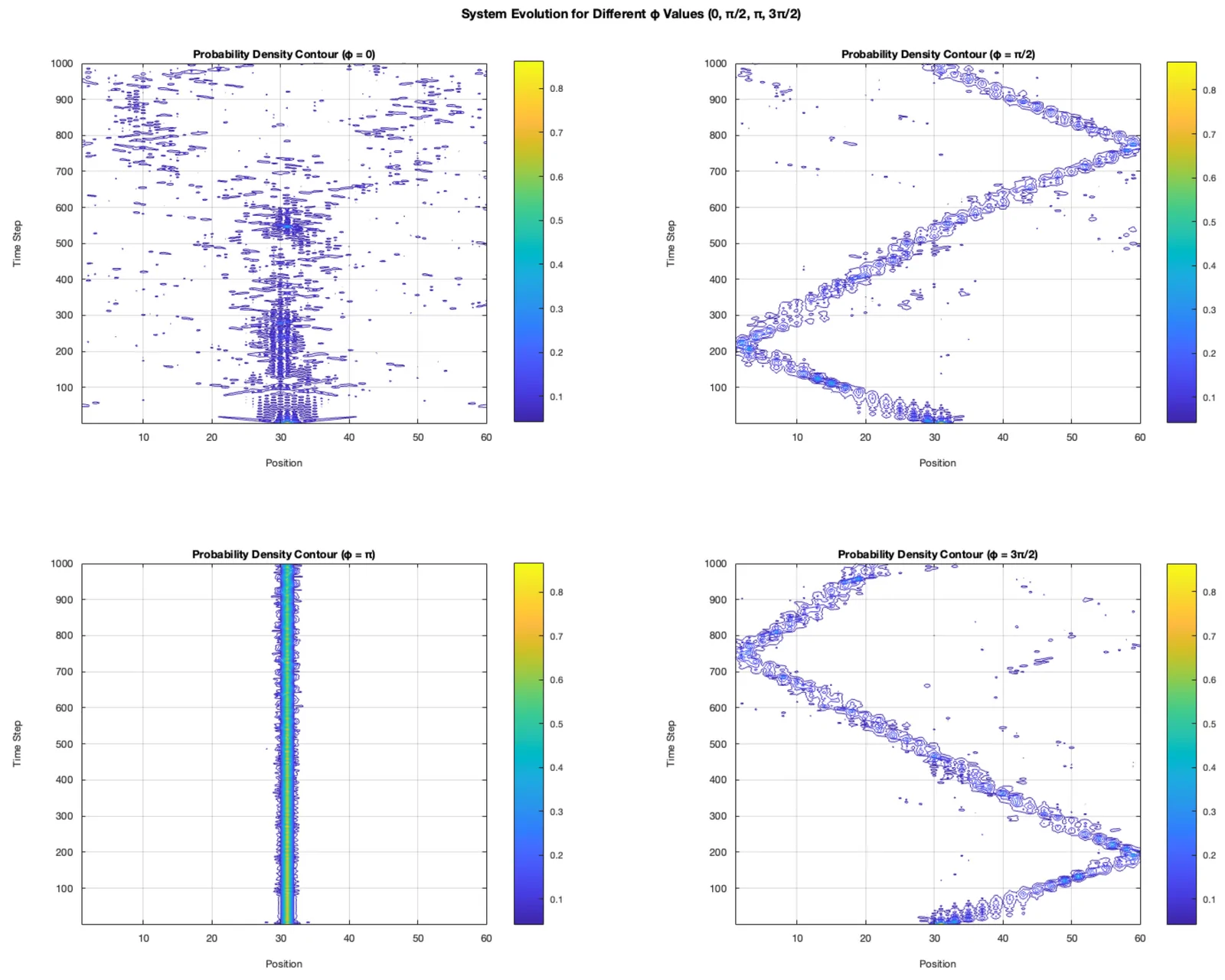
When the external magnetic flux takes different values, the relationship graph of the system probability density distribution evolution with time shows that the initial state of the system is at the thirty-first grid point. In this simulation, we set the number of grid points to 60. Considering that the intensity of the adjacent transition is greater than that of the secondary adjacent transition under normal circumstances, we set the intensity of the adjacent transition t to 2, the intensity of the secondary adjacent transition to 1, the interaction intensity u = 3.3, the single-particle dissipation intensity γ = 0, and the double-particle dissipation intensity κ = 0.

Now, from the formula, the intensities t of the nearest-neighbor transitions in the two directions are equal. Therefore, neither of them will dominate the direction of the system’s evolution. However, the next-nearest-neighbor hopping amplitude $t^{\prime }$ becomes crucial in determining the system evolution. Thus, a “seesaw” effect is formed. That is to say, when the intensity of the next-nearest-neighbor transition in one direction is relatively large, the initial evolution of the system will lean towards this direction and continue to evolve in this way. Let us take Equation (8) as an example. When the intensity of the next-nearest-neighbor transition in the j + 2 direction is less than that in the j − 2 direction, the system will have a tendency to transition towards the j − 2 direction in the initial state, as shown in the state when ϕ = 0.5π in Figure 2.

From Figure 2, we can see that since the external magnetic flux has a periodicity of 2π, this leads to the system evolution also having a periodicity of 2π. And when the external magnetic flux is a half-integer, that is, π/2 or 3π/2, the system evolution is directional. Therefore, in the subsequent evolution, we will uniformly take the external non-zero magnetic flux as π/2.

### 3.2. The Effect Produced by the Particle Transition t,t′

In the absence of particle dissipation, that is, γ = δ = κ = 0, the evolution of the system at this time is only related to the interaction and the transition between particles. From the analysis in the previous section, we know that if there were no next-nearest-neighbor transitions, the system would evolve simultaneously on both the left and right sides, and the evolutions on both sides would be equivalent. At this point, the evolution (Equation (7)) of the system becomes

(9) $$\frac{d}{dt}<{\hat{a}}_{j}>=it<{\hat{a}}_{j-1}>+it<{\hat{a}}_{j+1}>+2iu{\left|a_{j}\right|}^{2}<{\hat{a}}_{j}>$$

**Figure 3:**
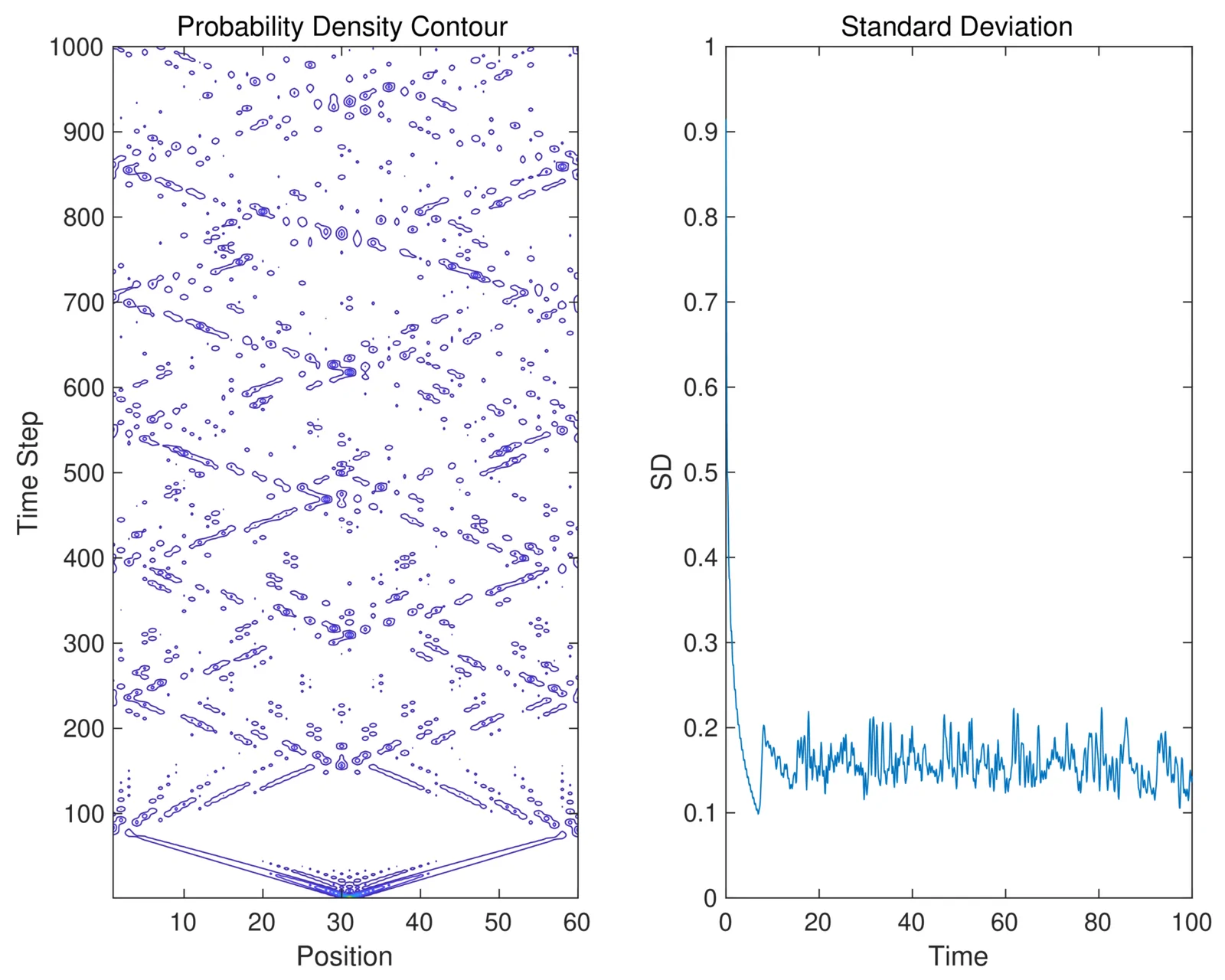
On the left is a schematic diagram of the evolution of the probability density of the system on the grid points over time, while on the right is a graph of the relationship between the standard deviation of the system and the evolution over time. In this simulation, the number of grid points we set remains 60. The intensity of the system’s near-transition t = 2, the interaction intensity u = 0.5, the external non-zero magnetic flux ϕ = π/2, and all other parameters are set to 0.

When the nearest-neighbor transition is dominant, the system will evolve to the left and right sides over time, and the trends on both sides are the same, as shown in Figure 3.

It can be seen that under the regulation of the external non-zero magnetic flux, the existence of nearest-neighbor transitions cannot cause the system to have directionality, while only next-nearest-neighbor transitions and the external non-zero magnetic flux can make the system have directionality. When the interaction becomes dominant, the self-trapping effect will occur in the system. Now, let us observe the dynamic equation of the system again:

(10) $$\begin{array}{cc} \frac{d}{dt}\langle {\hat{a}}_{j}\rangle & =-it^{\prime }\langle {\hat{a}}_{j+2}\rangle +it^{\prime }\langle {\hat{a}}_{j-2}\rangle +it\left(\langle {\hat{a}}_{j-1}\rangle +\langle {\hat{a}}_{j+1}\rangle \right)+2iU{\left|a_{j}\right|}^{2}\langle {\hat{a}}_{j}\rangle . \end{array}$$

**Figure 4:**
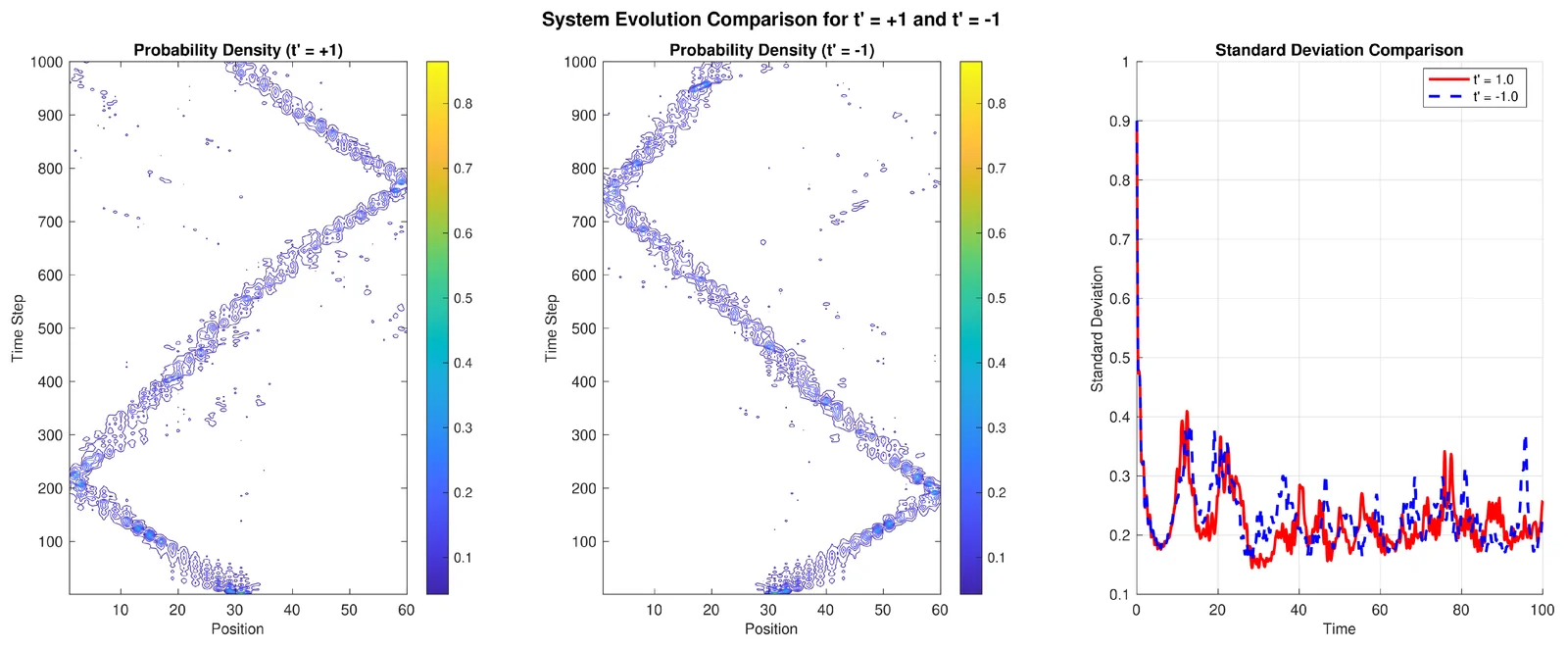
The left figure shows the evolution of the probability density on the grid points of the system over time, and the right figure is a schematic diagram of the transformation of the standard deviation of the system over time. In this simulation, we set the size of the system to 60, the interaction intensity u of the system to 3.3, and the intensity of the nearest-neighbor transition t to 2. In the left figure, we set the intensity of the next-nearest-neighbor transition to 1, and in the right figure, we set the intensity of the next-nearest-neighbor transition to −1.

Based on the previous discussion, we know that the next-nearest-neighbor transition t′ not only changes the law of system evolution, but also gives the transition direction through the regulation of magnetic flux. When the next-nearest-neighbor transition t′ is greater than 0, it can be observed that the evolution of the system has a tendency to move towards the grid point j − 2, that is, to evolve to the left. When we keep other parameters variable and set the next-nearest-neighbor transition as the opposite number, t = 2, t′ = −1, u = 3.3, then the system evolution direction at this time deflects as shown in Figure 4.

It can be seen that the evolution of the system only changes direction, but not the nature of the evolution. This result further indicates that the direction of the system evolution is the result of the combined effect of the next-nearest-neighbor transition intensity t′ and the external magnetic flux ϕ.

### 3.3. Particle Dissipation in the System (Single-Particle Dissipation Deviation γ±δ and Double-Particle Dissipation κ)

We know that in quantum mechanics, dissipation in a system is an inevitable process. When dissipation occurs, the particles on the lattice points will be excited to a higher-energy-level state. Therefore, we need to consider the influence of dissipation on the evolution of the system. First, consider the single-particle dissipation γ±δ of the system. At this time, the evolution equation of the system is

(11) $$\begin{array}{cc} \frac{d}{dt}\langle {\hat{a}}_{j}\rangle & =i\left[t^{\prime }\left(\langle {\hat{a}}_{j-2}\rangle -\langle {\hat{a}}_{j+2}\rangle \right)+t\left(\langle {\hat{a}}_{j-1}\rangle +\langle {\hat{a}}_{j+1}\rangle \right)\right]\\ \; & \;+i\left(2U{\left|a_{j}\right|}^{2}\right)\langle {\hat{a}}_{j}\rangle -\frac{1}{2}\left(\gamma +\delta \right)\langle {\hat{a}}_{j}\rangle . \end{array}$$

The odd and even positions in the system have different dissipation rates, which are γ−δ and γ+δ respectively. If both the dissipation γ and the deviation δ are positive, and the dissipation is greater than the deviation, that is, γ > δ, it will lead to the particle tending to flow from the even position to the odd position when the dissipation is strong at the even position. When dissipation is strong at odd positions, particles tend to flow from odd positions to even positions.

**Figure 5:**
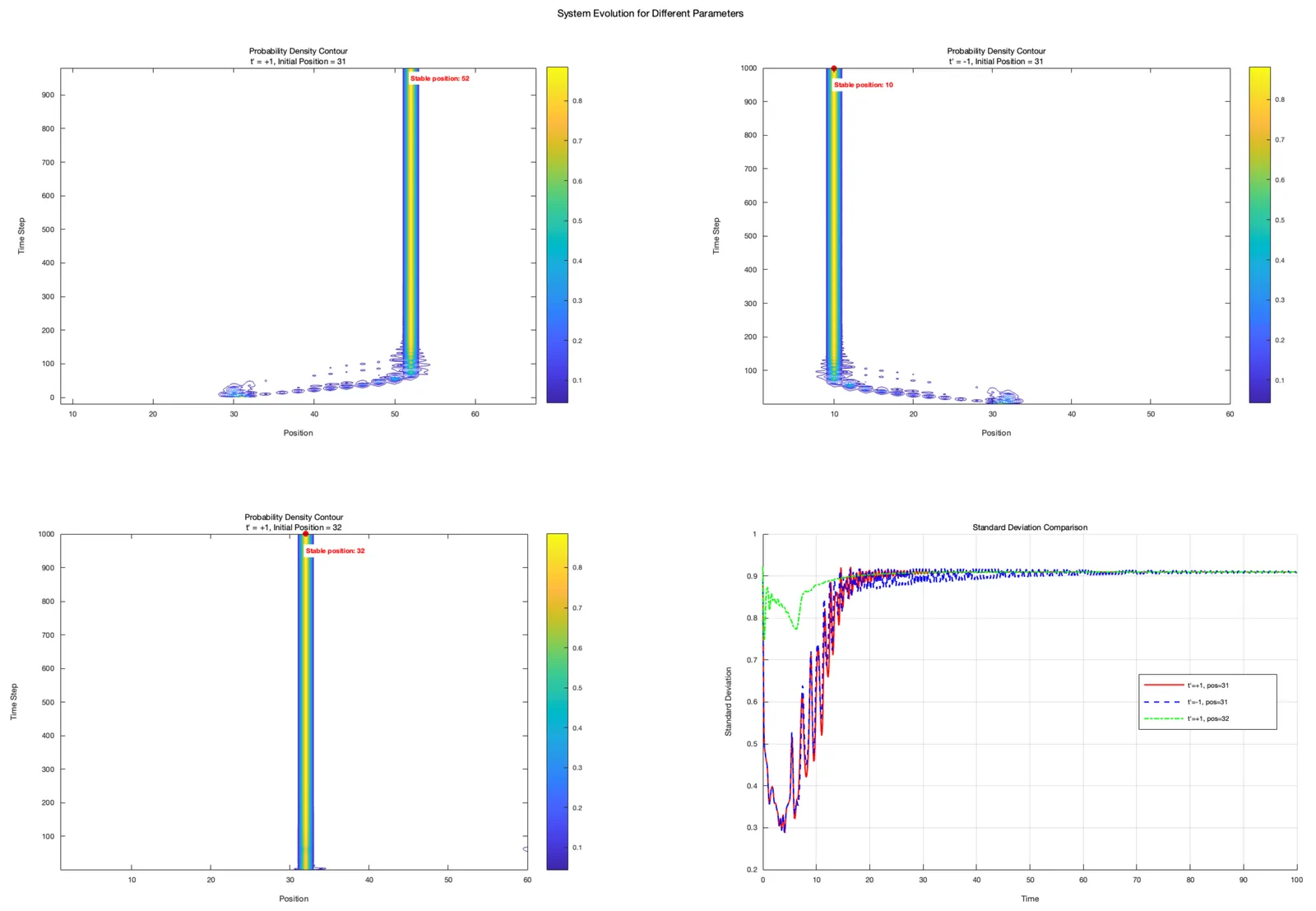
The diagram showing particle localization caused by dissipative deviation. System parameters: lattice size L=60, nearest-neighbor hopping t=2, on-site interaction U=6, single-particle dissipation γ=4, and staggered dissipation δ=2. The two figures above respectively illustrate the evolution patterns of the system’s initial state at odd grid points (here, we take 31) when t = 1 (on the left) and t = −1 (on the right). The figure on the left shows the evolution pattern of the system’s initial grid points at even grid points (here, we take 32) when t = 1. The right side presents the standard deviations for these three scenarios. Red dots in the left panels indicate stable lattice positions.

Take the initial state of the system at 31 grid points as an example. At this time, due to the existence of dissipation deviation, the dissipation at odd-numbered grid points is greater than that at even-numbered grid points. Then, the system will have a tendency to evolve towards even-numbered grid points. After a period of time, the system will remain stable at even grid points, as shown in Figure 5.

From the figure, we can observe that the dissipation is relatively high at the odd grid points, while it is lower at the even grid points. During the evolution of the system, it tends to localize at the grid points with lower dissipation, that is, at the even grid points. Moreover, after introducing an unbalanced dissipation deviation, the next-nearest-neighbor transition t′ still dominates the direction of system evolution. We also noticed that if the initial state of the system is at a grid point with lower dissipation, then on a macroscopic scale, the system will exhibit a localized state at the initial position. However, from the green curve of the standard deviation graph in Figure 5, we can see that although the system does not exhibit transport properties at this time, due to the combined effect of transitions and dissipation within the system, it will eventually stabilize after a period of time.

Now, taking the dissipation of two particles into account together, the evolution equation of the system at this time becomes

(12) $$\begin{array}{cc} \frac{d}{dt}\langle {\hat{a}}_{j}\rangle & =it^{\prime }e^{i\phi }\langle {\hat{a}}_{j+2}\rangle +it^{\prime }e^{-i\phi }\langle {\hat{a}}_{j-2}\rangle +it\left(\langle {\hat{a}}_{j-1}\rangle +\langle {\hat{a}}_{j+1}\rangle \right)\\ \; & \;+2iU{\left|a_{j}\right|}^{2}\langle {\hat{a}}_{j}\rangle -\frac{1}{2}\left(\gamma +\delta \right)\langle {\hat{a}}_{j}\rangle -\frac{\kappa }{2}{\left|a_{j}\right|}^{2}\langle {\hat{a}}_{j}\rangle . \end{array}$$

**Figure 6:**
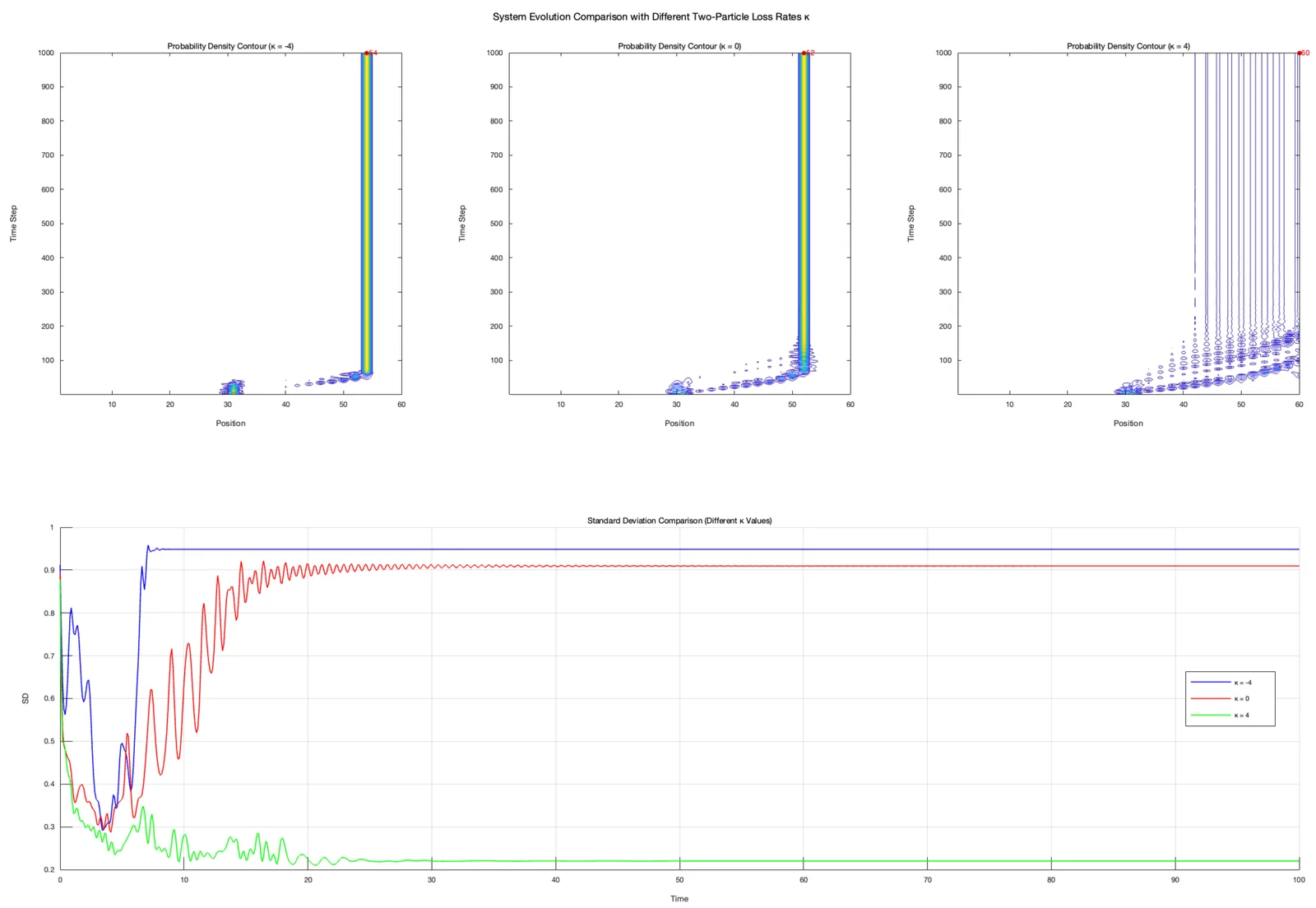
The left figure shows the evolution of the probability density of the system over time, and the right figure shows the evolution of the standard deviation of the system over time. The size of the system is set to 60. The initial state of the system is at the 31st grid point. The interaction intensity u = 6, the adjacent transition intensity t = 2, the secondary adjacent transition intensity t′ = 1, the single-particle dissipation γ = 4, and the dissipation deviation δ = 2.

Dissipation in quantum mechanics refers to the irreversible energy loss, coherence attenuation or information loss that occurs in a quantum system due to its interaction with the external environment (thermal reservoir). Dissipative channels (such as spontaneous emission and phonon coupling) provide additional transition paths for the system, accelerating energy relaxation. For two-particle dissipation, its influence is more obvious than that of single-particle dissipation. Therefore, compared with the system dispersion caused by single-particle dissipation, two-particle dissipation will destroy the original stability of the system and make the system more dispersed, as shown in Figure 6. And it also has a greater influence on the evolution speed of the system. If the two-particle dissipation is set to a negative κ < 0, the two-particle dissipation becomes the two-particle gain. When the two-particle gain is relatively large, the system will undergo a period of evolution and then reach an equilibrium state, as shown in Figure 6.

It can be seen that this result is similar to the situation without considering the dissipation of the two-particle system. The difference is that due to the influence of the two-particle gain, the system can reach the equilibrium state more quickly. Therefore, due to the deviation of single-particle dissipation at odd and even grid points in the system, the system will have a tendency to flow directionally towards odd or even numbers, and the introduction of two-particle gain will accelerate the evolution speed of the system.

## 4. Three-Site Reduced Lattice System

In the classic double-well optical system, we can observe many remarkable phenomena, such as the quantum tunneling effect and self-trapping effect. These phenomena usually occur when the system has a specific energy barrier structure and is influenced by the quantum interference effect. They reflect the nonlinear and complex dynamic behavior of the system under changes in external conditions. In our research, we will further expand this framework and assume that the research object is a non-equilibrium dissipative system with three optical lattices. Unlike the traditional double-well model, the configuration of the three optical lattices provides more abundant physical phenomena and dynamic characteristics, especially in terms of interaction and energy transfer. Moreover, our model also takes into account the influence of the external magnetic field, particularly the regulation of the external magnetic flux ϕ.

Based on our aforementioned discussion, the dynamic equations of the system have thus become

(13) $$\frac{da}{dt}=Ha+N\left(a\right)$$

with the matrix

(14) $$H=\left(\begin{array}{ccc} -\frac{\gamma +\delta }{2} & it & it^{\prime }e^{i\phi }\\ it & -\frac{\gamma -\delta }{2} & it\\ it^{\prime }e^{-i\phi } & it & -\frac{\gamma +\delta }{2} \end{array}\right)$$

and

(15) $$N\left(a\right)=\left(\begin{array}{c} \left(2iu-\frac{\kappa }{2}\right){\left|a_{1}\right|}^{2}\;a_{1}\\ \left(2iu-\frac{\kappa }{2}\right){\left|a_{2}\right|}^{2}\;a_{2}\\ \left(2iu-\frac{\kappa }{2}\right){\left|a_{3}\right|}^{2}\;a_{3} \end{array}\right)$$

where

$$a=\left(\begin{array}{c} \langle a_{1}\rangle \\ \langle a_{2}\rangle \\ \langle a_{3}\rangle \end{array}\right)$$

In order to further study the system dynamics under non-equilibrium dissipation, we assume that at this time an external non-zero magnetic flux of ϕ = π/2 is received, and γ = δ. The double-particle dissipation κ = 0. Thus, the dynamic equation becomes

(16) $$\frac{d}{dt}\left(\begin{array}{c} a_{1}\\ a_{2}\\ a_{3} \end{array}\right)=\left(\begin{array}{ccc} -\gamma & it & -t^{\prime }\\ it & 0 & it\\ t^{\prime } & it & -\gamma \end{array}\right)\left(\begin{array}{c} a_{1}\\ a_{2}\\ a_{3} \end{array}\right)+\left(\begin{array}{c} 2iu|a_{1}{|}^{2}a_{1}\\ 2iu|a_{2}{|}^{2}a_{2}\\ 2iu|a_{3}{|}^{2}a_{3} \end{array}\right).$$

At this point, we perform a semiclassical approximation for each lattice site, setting $a_{j}=\sqrt{n_{j}}e^{i\theta_{j}}$.

Additionally, we define the particle number difference operator and the relative phase difference operator as follows:

$$D=n_{1}+n_{3}-n_{2},\;\phi_{D}=\theta_{1}+\theta_{3}-2\theta_{2}.$$

Thus, we can obtain the following relationship equation:

(17) $$\begin{array}{cc} \dot{D} & =-4t\left[\sqrt{n_{1}n_{2}}\sin\left(\theta_{2}-\theta_{1}\right)+\sqrt{n_{3}n_{2}}\sin\left(\theta_{2}-\theta_{3}\right)\right]-2\gamma \left(n_{1}+n_{3}\right), \end{array}$$

(18) $$\begin{array}{cc} {\dot{\phi }}_{D} & =-\frac{n_{1}+n_{3}}{\sqrt{n_{1}n_{3}}}\sin\left(\theta_{3}-\theta_{1}\right)+t\left[\sqrt{\frac{n_{2}}{n_{1}}}\cos\left(\theta_{2}-\theta_{1}\right)+\sqrt{\frac{n_{2}}{n_{3}}}\cos\left(\theta_{2}-\theta_{3}\right)\right.\\ \; & \;\left.-2\sqrt{\frac{n_{1}}{n_{2}}}\cos\left(\theta_{1}-\theta_{2}\right)-2\sqrt{\frac{n_{3}}{n_{2}}}\cos\left(\theta_{3}-\theta_{2}\right)\right]+2UD. \end{array}$$

We assume that when the system reaches a stable state, the relationship between the particle number difference and time changes approaches 0, i.e., $\dot{D}\approx 0$. Moreover, due to the symmetry of the system, we can assume that $n_{1}=n_{3}=n$, and define the following angle differences:

$$\phi_{1}=\theta_{2}-\theta_{1},\;\phi_{2}=\theta_{2}-\theta_{3}.$$

Then, the dynamic equation of the system is

(19) $$0=-4t\sqrt{n(N_{\mathrm{tot}}-2n)}\left[\sin\phi_{1}+\sin\phi_{2}\right]-4\gamma n$$

If the phase differences throughout the entire lattice remain uniformly consistent, $\phi_{1}=\phi_{2}=\phi$.

(20) $$2\sin\phi =-\frac{\gamma n}{t\sqrt{n(N_{\mathrm{tot}}-2n)}}$$

(21) $$\phi_{D}=-2arcsin\left(\frac{\gamma (D+N_{tot})}{4t\sqrt{\left(D+N_{tot}\right)\left(N_{tot}-D\right)}/2}\right)$$

**Figure 7:**
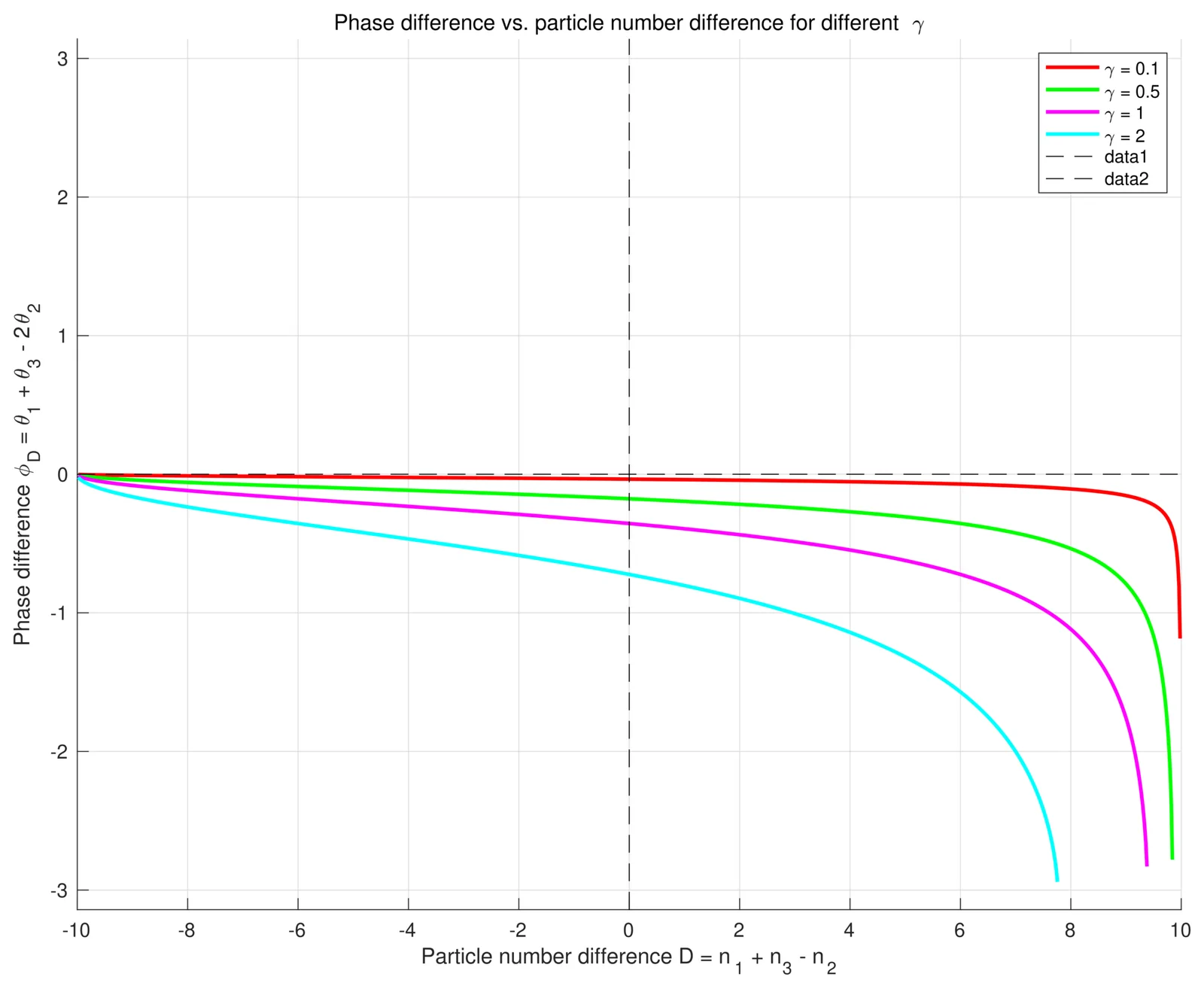
Graph showing the relationship between the phase difference and the particle number difference of the system under different dissipative deviations γ. The total particle number of the system is set to $N_{\mathrm{tot}}=10$.

As can be seen from Figure 7, at lower dissipation deviations (such as γ = 0.1), the influence of the particle number difference is relatively small, and the phase difference of the system changes more smoothly, indicating that at this time the phase difference of the system is not strongly affected by the particle number difference. At higher dissipation deviations (such as γ = 2.0), the change in the phase difference is larger, suggesting that the system responds more vigorously to the particle number difference because the stronger dissipation deviation leads to more energy or information loss, making the change in the phase difference more sensitive. At the same time, a larger deviation also causes a more intense localized degree at even-numbered lattice points.

## 5. Conclusions

In conclusion, in this study, we investigated the dynamics of cold atom dissipation models with non-Hermitian Hamiltonian quantities in optical lattices. The influence of dissipation and transition on non-Hermitian systems is explored through numerical simulation and analysis. The research results show that the introduction of external non-zero magnetic flux has the ability to regulate the evolution direction of non-Hermitian systems. This effect is caused by the interaction between non-zero magnetic flux and next-nearest-neighbor transitions. Not only that, in our research, non-uniform single-particle dissipation was also introduced. The simulation results show that due to the different dissipation intensities of odd and even positions, the final local lattice points of the system evolution are also different. If the dissipation intensity at the odd-numbered grid points is greater than that at the even-numbered grid points, then the evolution of the system will eventually flow from the odd-numbered grid points to the even-numbered grid points, and vice versa. Finally, we discussed the influence of two-particle dissipation on the evolution of the system. The results show that uniform two-particle dissipation makes the system more dispersed. However, if the two-particle dissipation becomes negative, that is, if the two-particle dissipation becomes gain, then the evolution speed of the system will increase.

## Data Availability

The original contributions presented in this study are included in the article. Further inquiries can be directed to the corresponding author.
